# National survey on the availability of oncology palliative care services at tertiary general and cancer hospitals in China

**DOI:** 10.1186/s12904-023-01259-5

**Published:** 2023-09-28

**Authors:** Xiaomei Li, Xin Shelley Wang, Haili Huang, Miao Liu, Yinan Wu, Jiaojiao Qiu, Boran Zhang, Linhong Cui, David Hui

**Affiliations:** 1 Department of Geriatric Medicine, The Second Medical Center, Chinese PLA General Hospital, Haidian District, 28Th, Fuxing Road, Beijing, 100853 People’s Republic of China; 2 Department of Symptom Research, The University of Texas MD Anderson Cancer Center, Houston, TX USA; 3 Department of Medical Oncology, The Second Medical Center, Chinese PLA General Hospital, Beijing, People’s Republic of China; 4 Graduate School, Chinese PLA General Hospital, Beijing, People’s Republic of China; 5 Publicity Department, Beijing Hospital, Beijing, People’s Republic of China; 6 Department of Palliative Care, Rehabilitation and Integrative Medicine, The University of Texas MD Anderson Cancer Center, Houston, TX USA

**Keywords:** Palliative care, Availability, Cancer, National survey, Tertiary general hospital, China

## Abstract

**Background:**

This nationwide survey studied the level of palliative care (PC) access for Chinese patients with cancer among cancer care providers either in tertiary general hospitals or cancer hospitals in China.

**Methods:**

Using a probability-proportionate-to-size method, we identified local tertiary general hospitals with oncology departments to match cancer hospitals at the same geographic area. A PC program leader or a designee at each hospital reported available PC services, including staffing, inpatient and outpatient services, education, and research, with most questions adapted from a previous national survey on PC. The primary outcome was availability of a PC service.

**Results:**

Most responders reported that some type of PC service (possibly called “comprehensive cancer care,” “pain and symptom management,” or “supportive care”) was available at their institution (84.3% of tertiary general hospitals, 82.8% of cancer hospitals). However, cancer hospitals were significantly more likely than tertiary general hospitals to have a PC department or specialist (34.1% vs. 15.5%, *p* < 0.001). The most popular services were pain consultation (> 92%), symptom management (> 77%), comprehensive care plans (~ 60%), obtaining advanced directives and do-not-resuscitate orders (~ 45%), referrals to hospice (> 32%), and psychiatric assessment (> 25%). Cancer hospitals were also more likely than tertiary general hospitals to report having inpatient beds for PC (46.3% vs. 30.5%; *p* = 0.010), outpatient PC clinics (28.0% vs. 16.8%; *p* = 0.029), educational programs (18.2% vs. 9.0%, *p* = 0.014), and research programs (17.2% vs. 9.3%, *p* < 0.001).

**Conclusions:**

Cancer hospitals are more likely to offer PC than are tertiary general hospitals in China. Our findings highlight opportunities to further increase the PC capacity in Chinese hospitals.

**Supplementary Information:**

The online version contains supplementary material available at 10.1186/s12904-023-01259-5.

## Introduction

China has an aging population and a rapidly increasing number of patients diagnosed with advanced cancer [1], resulting in a growing need for palliative care (PC)-relevant specialized professionals and education [2, 3]. Palliative care was initially introduced to China in the early 1990s, starting with conceptual training on cancer pain and symptom management for oncology professionals [4], followed by the establishment of national policies to increase the availability of opioids and mandated professional education for basic cancer pain management. The Chinese Ministry of Health published a guideline on pain management in 2011, and the Good Pain Management program was launched that year to standardize care [5]. The National Health Commission issued a series of official policies and regulations on PC and launched the first wave of national hospice care pilot centers in 2017 [6]. Despite these efforts, a recent multicenter study found that the availability of oncology PC services in China was suboptimal, with several significant factors contributing to this lack [7]. Moreover, the availability of PC programs and PC providers from Chinese academic institutions, both primary oncologists and PC specialists, and the infrastructure necessary to support them, are unclear [8].

A better understanding of current PC practice in China would inform public health policy, resource allocation, and program planning for government officials, hospital administrators, and clinicians. We therefore conducted a national survey to compare the availability of PC in tertiary general hospitals with that in cancer hospitals in China. We also investigated the extent of PC education and research at these hospitals.

## Methods

### Survey development

The primary outcome of this study was availability of a PC service at each hospital. Survey questions were based on those used in similar multicenter studies conducted in the United States in 2010 and 2020 [8, 9] focused on aspects of PC provision at each hospital, such as personnel comprising the PC team, scope of service, structure and processes of the PC program (if available), and educational and research activities. An additional 7 questions were added to tailor the surveys to current PC practice in China (see Supplement). A cognitive debriefing on meaningfulness of the English-to-Chinese translation of the original survey questions resulted in minor modification of the survey wording. The term *palliative care* was defined at the beginning of the survey as an active service aimed at reducing physical and mental suffering in persons with advanced cancer or another incurable illness.

The Institutional Review Board at The Chinese PLA General Hospital approved the study and waived the requirement for informed consent.

### Sampling method and sample size

The nationwide survey covered 31 of China’s 32 regions (22 provinces, 5 autonomous regions, and 4 municipalities directly under the Central Government); we excluded the Xizang (Tibet) autonomous region, because it has no recognized medical oncology care. The targeted hospitals were government-registered tertiary general hospitals and cancer hospitals, where most cancer patients in China receive care. The National Health Commission of the People’s Republic of China accredits hospitals as tertiary general hospitals on the basis of standards that encompass a range of clinical, research, and quality-improvement aspects of health care; these hospitals often have at least 500 beds. A cancer hospital is defined as a center dedicated to the diagnosis and treatment of patients with cancer.

We surveyed all cancer hospitals listed in the national registry. For tertiary general hospitals, we used the probability-proportionate-to-size method to determine the appropriate sample size [10]. In 2019, 1,981 tertiary general hospitals on the national registry were providing oncology care to adult patients. Assuming an availability rate of 60% for tertiary general hospitals, we calculated that at least 257 hospitals would need to be surveyed to achieve 80% power to test the primary outcome with a two-sided confidence interval width at 0.12 and alpha at 0.05. Assuming a 40% nonresponse rate, a sample of 480 tertiary general hospitals was appropriate.

### Survey administration

To identify a PC leader at each hospital, we searched either the institution’s website or a list of PC providers active in academic activities, and confirmed with the hospital. If no assigned PC leader could be identified, a leading medical oncologist or their recommended personnel answered the survey.

Trained volunteers contacted the identified individuals to describe the study and invite them to participate. The survey was administered through a secured website (https://jinshuju.net/) and could be completed on a personal device electronically and submitted immediately. Nonresponders received a telephone reminder after 1–2 weeks. If no response, research staff visited the hospital in person to administer the survey. Data were stored on the King data platform (https://jinshuju.net/forms/).

### Statistical analysis

We used standard descriptive statistics, including medians, inter-quartile ranges, proportions, and frequencies, together with 95% CIs where appropriate, to summarize the availability of various types of PC services at different hospitals. Differences in services provided between the two types of hospitals were computed by using the Fisher exact test for selected categorical variables and the Mann–Whitney test for selected non-parametric continuous variables; *p* < 0.05 was considered statistically significant. The Statistical Package for the Social Sciences (SPSS version 16.0, SPSS Inc., Chicago, Illinois) software was used for statistical analysis.

## Results

### Sample characteristics

The study was initiated in June 2019 and completed in February 2020. The final analysis included 99 cancer hospitals (48.1% of all 206 cancer hospital nationwide) and 268 general hospitals (60.5% of all 443 government-registered tertiary general hospitals that defined by probability-proportionate-to-size matched method).

Table 1 presents the characteristics of hospitals that participated in the study. Significantly more cancer hospitals than tertiary general hospitals were privately owned (32.3% compared with 4.5%; *p* < 0.001). Almost all hospitals were geographically located in metropolitan areas; 45 participating cancer hospitals (45.5%) were considered non-tertiary hospitals.

**Table 1:** Characteristics of Chinese hospitals participating in the study

	**Tertiary general hospitals (*n* = 268)**		**Cancer hospitals (*n* = 99)**		***p* value**
	**n**	**% (95%CI)**	**n**	**% (95%CI)**	
Region					0.315
Eastern	124	46.3 (40.3–52.3)	47	47.5 (37.8–57.3)	
Central	71	26.5 (21.5–32.1)	32	32.3 (23.8–42.2)	
Western	73	27.2 (22.2–32.9)	20	20.2 (13.4–29.3)	
Public or private hospital					< 0.001
Public	256	95.5 (92.2–97.4)	67	67.7 (57.8–76.2)	
Private	12	4.5 (2.6–7.7)	32	32.3 (23.8–42.1)	
Type of hospital					< 0.001
Tertiary	268	100.0	54	54.5 (2.1–11.6)	
Non-tertiary	0	0	45	45.5 (35.9–55.3)	
Palliative care service available					0.728
Yes	226	84.3 (79.9–88.7)	82	82.8 (75.3–90.4)	
No	42	15.7	17	17.2	

### Availability of palliative care

Most hospitals in mainland China reported having some level of PC available (*n* = 226/268 general hospitals, 84.3%; *n* = 82/99 cancer hospitals, 82.8%). See Table 1.

Table 2 presents characteristics of the various PC services available at hospitals participating in our study. A significantly greater proportion of cancer hospitals than tertiary general hospitals reported having a PC department or specialist (34.1% vs. 15.5%, *p* < 0.001). Most respondents (> 85%) were senior-level faculty, and most respondents reported working in a medical oncology department (81.9% of general hospital and 59.8% of cancer hospital respondents, *p* = 0.029).

**Table 2:** Characteristics of PC programs offered at Chinese hospitals participating in the study

	**Tertiary general hospitals (*n* = 226)**		**Cancer hospitals (*n* = 82)**		***p* value**
	***n***	**% (95%CI)**	***n***	**% (95%CI)**	
PC service offered by:					0.001
PC department or specialists	35	15.5 (10.7–20.2)	28	34.1 (25.0–45.6)	0.0006
Other professionals (no PC department or specialists)	183	81.0 (75.8–86.1)	51	62.2 (51.9–72.8)	
Missing	8	3.5 (1.1–6.0)	3	3.7 (0.6–7.8)	
Title of PC program (multiple answers allowed)					
Comprehensive cancer care	167	73.9(68.1–79.7)	52	63.4(52.8–74.1)	0.073
Pain and symptom management	141	62.4 (56.0–68.8)	46	56.1 (45.1–67.1)	0.318
Palliative care	54	23.9 (18.3–29.5)	20	24.4 (14.9–33.9)	0.928
End-of-life care	76	33.6 (27.4–39.8)	28	34.1 (23.7–44.6)	0.932
Supportive care	128	56.6 (50.1–63.1)	34	41.5 (30.6–52.4)	0.018
Time since initiation of PC program					0.746
< 1 year	17	7.5 (4.1–11.0)	7	8.5 (4.1–16.9)	
1–2 years	38	16.8 (12.4–22.3)	16	19.5 (12.3–29.6)	
3–5 years	51	22.6 (17.6–28.5)	16	19.5 (12.3–29.6)	
> 5 years	120	53.1 (46.5–59.5)	43	52.4 (41.6–63.0)	
Respondent’s professional title					0.142
Senior faculty member	210	92.9 (89.6–96.3)	71	86.6 (79.2–94.0)	
Mid-level faculty member	15	6.6 (3.4–9.9)	11	13.4 (6.0–20.8)	
Junior faculty member	1	0.4 (− 0.4 - 1.3)	0	0	
At least 20% of PC staff are full-time physicians	82	36.3 (30.0–42.6)	36	43.9 (33.2–54.6)	0.224
Respondent’s department within hospital					0.029
Medical oncology	185	81.9 (76.2–86.3)	49	59.8 (48.8–69.8)	0.000
Medical department	7	3.1 (1.5–6.4)	6	7.3 (3.3–15.4)	
Radiology	6	2.7 (1.2–5.7)	7	8.5 (4.1–16.9)	
Traditional Chinese Medicine	4	1.8 (0.7–4.6)	2	2.4 (0.6–9.3)	
Pain management	4	1.8 (0.6–4.6)	5	6.1 (2.6–13.9)	
Geriatric care	4	1.8 (0.7–4.6)	2	2.4 (0.6–9.3)	
Surgery	3	1.3 (0.4–4.1)	3	3.7 (1.2–10.8)	
Nursing	3	1.3 (0.4–4.1)	2	2.4 (0.6–9.3)	
Palliative care	2	0.9 (0.2–3.4)	1	1.2 (0.2–8.2)	
Other	8	3.5 (0.2–6.9)	5	6.1 (2.6–13.9)	
PC services provided (multiple answers allowed)					
Assessment and management of psychiatric disorders	87	38.5 (32.2–44.8)	21	25.6 (16.2–35.1)	0.036
Obtaining advanced directives/DNR orders	102	45.1 (38.6–51.6)	37	45.1 (34.4–55.9)	0.999
Solving ethical issues	51	22.6 (17.1–28.0)	23	28.0 (18.3–37.8)	0.320
Referrals to hospice, home care, and other placements	72	31.9 (25.8–37.9)	36	43.9 (33.2–54.6)	0.050
Comprehensive care plans	134	59.3 (52.9–65.7)	49	59.8 (49.1–70.4)	0.942
Pain consultation	212	93.8 (90.7–96.9)	76	92.7 (87.0–98.3)	0.724
Psychosocial support	114	50.4 (43.9–57.0)	38	46.3 (35.5–57.1)	0.525
Symptom management	174	77.0 (71.5–82.5)	64	78.0 (69.1–87.0)	0.845
Estimated patient follow-up time					0.109
1-7 days	43	19.0 (13.9–24.1)	12	14.6 (7.0–22.3)	
1-4 weeks	76	33.6 (27.5–39.8)	23	28.0 (18.3–37.8)	
1-12 months	76	33.6 (27.5–39.8)	31	37.8 (27.3–48.3)	
1-2 years	17	7.5 (4.1–11.0)	9	11.0 (4.2–17.7)	
> 2years	14	6.2 (3.1–9.3)	7	8.5 (2.5–14.6)	

*DNR* Do not resuscitate, *PC* palliative care

Most PC services were called “comprehensive cancer care,” followed by “pain and symptom management” or “supportive care,” and this was consistent across tertiary general hospitals and cancer hospitals. About one-third of each type of hospital also referred to PC as “end-of-life care.” More than half of all hospitals had offered a PC service for at least 5 years. Approximately 36% of tertiary general hospitals and 44% of cancer hospitals reported that at least 20% of their staff providing PC were full-time physicians.

A wide range of PC services were provided by both types of hospitals; the most popular services were pain consultation (> 92%), symptom management (> 77%), comprehensive care plans (~ 60%), obtaining advanced directives and do-not-resuscitate (DNR) orders (~ 45%), referrals to hospice (> 32%), and psychiatric assessment (> 25%). About one-third of all hospitals reported patient follow-up times within 1 month, approximately one-third reported follow-up times within 1 year, and approximately 15% reported follow-up times of more than 1 year.

### Structures and processes of clinical palliative care services in China

Table 3 presents the structures and processes of PC programs in the participating hospitals.

**Table 3:** Structures and processes of PC inpatient and outpatient clinical services in Chinese hospitals participating in the study

	**Tertiary general hospitals (*n* = 226)**		**Cancer hospitals (*n* = 82)**		***p* value**
	**n**	**% (95% CI)**	**n**	**% (95% CI)**	
PC inpatient beds, with or without dedicated PC department					0.010
Yes	69	30.5 (24.5–36.5)	38	46.3 (35.5–57.1)	
No	157	69.5 (63.5–75.5)	44	53.7 (42.5–64.5)	
Hospitals with inpatient PC beds	69		38		
Mean number of beds	30.6		19.7		0.028
Mean time since initiation of PC program					
> 3years	49	71.0	20	52.6	0.060
Reasons for admission to inpatient PC unit, median % [IQR]					
Dying	20.0 [10.0–50.0]		20.0 [5.0–60.0]		0.817
Urgent symptom control	20.0 [10.0–40.0]		20.0 [5.0–50.0]		0.647
Psychosocial issue	6.0 [3.8–10.0]		5.0 [0.0–11.5]		0.427
Family’s inability to continue to care for the patient	10.0 [5.0–20.0]		5.0 [0.0–12.5]		0.144
Other reason	5.0 [1.0–10.0]		4.0 [0.0–29.5]		0.562
Proportion of PC inpatients receiving regular psychosocial assessment					0.647
0%	8	11.6 (4.0–19.1)	6	15.8 (4.2–27.4)	
1–25%	28	40.6 (29.0–52.2)	12	31.6 (16.8–46.4)	
26–100%	33	47.8 (35.7–59.9)	20	52.6 (36.0–69.3)	
Family meeting during inpatient PC					0.831
0%	7	10.1 (3.0–17.3)	3	7.9 (− 0.7–16.5)	
1–25%	35	50.7 (38.9–62.5)	20	52.6 (36.8–68.5)	
26–50%	11	15.9 (7.3–24.6)	8	21.1 (8.1–34.0)	
51–75%	8	11.6 (4.0–19.1)	5	13.2 (2.4–23.9)	
76–100%	8	11.6 (4.0–19.1)	2	5.3 (− 1.8–12.4)	
Proportion of inpatients with DNR orders					0.275
0%	4	5.8 (0.3–11.3)	3	7.9 (− 0.7–16.5)	
1–25%	13	18.8 (9.6–28.1)	12	31.6 (16.8–46.4)	
26–50%	13	18.8 (9.6–28.1)	2	5.3 (− 1.8–12.4)	
51–75%	12	17.4 (8.4–26.3)	6	15.8 (4.2–27.4)	
76–100%	27	39.1 (27.6–50.6)	15	39.5 (23.9–55.0)	
PC consultations for inpatients available					0.492
Yes	103	45.6 (39.1–52.1)	41	50.0 (39.2–60.8)	
No	123	54.4 (47.9–60.9)	41	50.0 (39.2–60.8)	
Hospitals offering inpatient PC consultation	103		41		
Availability of consultations					0.770
24 h/day, 7 days/week	36	35.0 (25.7–44.2)	17	41.5 (26.4–56.5)	
24 h/day, weekdays only	13	12.6 (6.2–19.0)	3	7.3 (− 0.7–15.3)	
8 h/day, weekdays only	48	46.6 (37.0–56.2)	19	46.3 (31.1–61.6)	
Other	6	5.8 (1.3–10.3)	2	4.9 (− 1.7–11.5)	
Median referrals per month, n [IQR]	10.0 [5.0–15.0]		10.0 [5.0–20.0]		0.440
Median days from referral to death, n [IQR]	50.0 [30.0–90.0]		60.0 [30.0–117.5]		0.434
Department or unit requesting consultation					
Emergency department	34	33.0 (23.9–42.1)	12	29.3 (15.3–43.2)	
Medical oncology	86	83.5 (76.3–90.7)	39	95.1 (88.5–101.7)	
Surgery	59	57.3 (47.7–66.8)	29	70.7 (56.8–84.7)	
Hematology	43	41.7 (32.2–51.3)	12	29.3 (15.3–43.2)	
Radiation oncology	48	46.6 (37.0–56.2)	28	68.3 (54.0–82.5)	
Other	31	30.1 (21.2–39.0)	5	12.2 (2.2–22.2)	
Outpatient PC clinic available					0.029
Yes	38	16.8 (11.9–21.7)	23	28.0 (18.3–37.8)	
No	188	83.2 (78.3–88.1)	59	72.0 (62.2–81.7)	
Hospitals with an outpatient PC clinic	38		23		
Type of outpatient PC clinic:					0.004
Dedicated palliative care outpatient clinic	6	15.8 (4.2–27.4)	6	26.1 (8.1–44.0)	
Oncology clinic	28	73.7 (59.7–87.7)	7	30.4 (11.6–49.2)	
Other	4	10.5 (0.8–20.3)	10	43.5 (23.2–63.7)	
PC clinic days/week, n		5.0 (3.0–5.0)		5.0 (2.0–5.0)	0.800
Patents visiting PC clinic/month, n		50.0 (20.0–175.0)		100.0 (20.0–300.0)	0.322
Time from first PC clinic visit to death, days		85.0 (46.5–97.5)		60.0 (30.0–135.0)	0.922
Patient referred to PC clinic from:					
Emergency department	14	36.8 (21.5–52.2)	8	34.8 (15.3–54.2)	0.871
Medical oncology	36	94.7 (87.6–101.8)	21	91.3 (79.8–102.8)	0.600
Surgery	13	34.2 (19.1–49.3)	11	47.8 (27.4–68.2)	0.291
Hematology	17	44.7 (28.9–60.5)	4	17.4 (1.9–32.9)	0.029
Radiation oncology	13	34.2 (19.1–49.3)	11	47.8 (27.4–68.2)	0.291
Patient self	33	86.8 (76.1–97.6)	18	78.3 (61.4–95.1)	0.380
Other	7	18.4 (6.1–30.7)	4	17.4 (1.9–32.9)	0.919

*DNR* do not resuscitate, *IQR* inter quartile range, *PC* palliative care

#### Inpatient palliative care

More cancer hospitals than tertiary general hospitals provided inpatient beds for their PC programs, regardless of the availability of a dedicated PC department (46.3% compared with 30.5%; *p* = 0.010); an average of 31 beds were available for PC at tertiary general hospitals, compared with 20 beds at cancer hospitals. Most hospitals with inpatient PC beds had offered PC for more than 3 years (71.0%of tertiary general hospitals and 52.6% of cancer hospitals).

The most common reasons for admission to the inpatient PC unit included expected death (median 20%), urgent symptom control needs (median 20%), psychosocial issues (median 5%-6%), and lack of caregivers (median 5%-10%). In about half of hospitals, at least 1 in 4 inpatients receiving PC underwent regular psychosocial evaluation, although few (1%-25%) had regular family meetings with professionals. However, the practice of having DNR orders was quite common, with approximately 40%of hospitals reporting that 75%-100% of PC inpatients had DNR orders.

#### Palliative care consultation

Among hospitals offering PC consultation for inpatients (45.6% of tertiary general hospitals and 50.0% of cancer hospitals), about 60% of hospitals offered PC consultations only on weekdays, and more than one-third of hospitals also offered consultations on weekends (35.0% of tertiary general hospitals and 41.5% of cancer hospitals), with a median referral rate of 10 patients per month. The median time from PC consultation to death was 50 days in tertiary general hospitals and 60 days in cancer hospitals. Although medical oncology departments were the most common units requesting a PC consultation, other units commonly requesting consultations included surgery, the emergency department, and hematology. Cancer hospitals reported a higher rate of consultation requests from radiation oncology than did tertiary general hospitals (68.3% compared with 46.6%; *p* = 0.019).

#### Outpatient palliative care

The availability of outpatient PC clinics in China was limited; however, more cancer hospitals than tertiary general hospitals provided PC outpatient clinics (28.0% compared with 16.8%; *p* = 0.029). In tertiary general hospitals, outpatient PC clinics were more often part of the medical oncology clinic (73.7%) than offered as a dedicated PC clinic (15.8%); in contrast, in cancer hospitals, 30.4% of outpatient PC clinics were part of medical oncology, 26.1% were dedicated outpatient PC clinics, and 43.5% were offered by other professionals. A median 50–100 patients per month received PC outpatient consultations, often offered only on weekdays. The median time from first PC consultation to patient death was approximately 85 days in tertiary general hospitals and 60 days in cancer hospitals. Most patients receiving outpatient PC consultations were referred from medical oncology departments (94.7% in tertiary general hospitals, 91.3% in cancer hospitals) or self-referred (86.8% and 78.3%, respectively), followed by referrals from hematology, the emergency department, surgery, and radiation oncology.

#### Palliative care education and research in Chinese hospitals

PC education and research characteristics for the hospitals participating in our study are summarized in Table 4. Palliative care educational programs for clinical or research fellows were offered by 9.0% of tertiary general hospitals and 18.2% of cancer hospitals (*p* = 0.014). Grand rounds focused on PC were offered at 24.6% of tertiary general hospitals and 33.3% of cancer hospitals overall; in contrast, more than 97% were offered specifically to oncologists.

**Table 4:** Status of PC education and research in Chinese hospitals participating in the study

	**Tertiary general hospitals (*n* = 268)**		**Cancer hospitals (*n* = 99)**		***p* value**
	***n***	**% (95%CI)**	***n***	**% (95%CI)**	
PC training program available					0.014
Yes	24	9.0 (5.5–12.4)	18	18.2 (10.6–25.8)	
No	244	91.0 (87.6–94.5)	81	81.8 (74.2–89.4)	
Professional requirement					
PC board certification for physicians	41/226	18.1 (13.1–23.2)	16/82	19.5 (10.9–28.1)	0.784
PC board certification for nurses	36/226	15.9 (11.2–20.7)	18/82	22.0 (13.0–30.9)	0.219
Use of patient-reported outcomes in hospitals offering PC	90/226	39.8(33.4–46.2)	37/82	45.1 (34.4–55.9)	0.404
PROM-NRS pain scale	75/226	33.2 (27.0–39.3)	32/82	39.0 (28.5–49.6)	0.342
PROM-multidimensional scale (BPI)	57/226	25.2 (19.6–30.9)	25/82	30.5 (28.5–49.6)	0.355
PROM-multi symptom assessment (MDASI)	37/226	16.4 (11.5–21.2)	13/82	15.9 (7.9–23.8)	0.913
PC scale (ESAS)	25/226	11.1 (7.0–15.2)	10/82	12.2 (5.1–19.3)	0.782
PC topics offered at grand rounds					0.095
Yes	66	24.6 (19.5–29.8)	33	33.3 (24.0–42.6)	
No	202	75.4 (70.2–80.5)	66	66.7 (57.4–76.0)	
Hospitals offering PC grand rounds	66		33		0.999
PC grand rounds offered to oncologists	65	98.5 (95.5–101.4)	32	97.0 (91.1–102.8)	
PC research projects available					< 0.001
Yes	25	9.3 (5.8–12.8)	17	17.2 (9.7–24.6)	
No	243	90.7 (87.2–94.2)	82	82.8 (75.4–90.3)	
Among hospitals with research projects available	25		17		
Receives research funding					0.913
Yes	5	20.0 (4.3–35.7)	4	23.5 (3.4–43.7)	
No	20	80.0 (64.3–95.7)	13	76.5 (56.3–96.6)	
PC research conducted in the past year					
Prospective therapeutic RCT	3	12.0 (− 0.7–24.7)	5	29.4 (7.8–51.1)	0.312
Prospective non-therapeutic study	3	12.0 (− 0.7–24.7)	1	5.9 (− 5.3–17.1)	0.899
Other prospective study	3	12.0 (− 0.7–24.7)	7	41.2 (17.8–64.6)	0.070
Retrospective study	14	56.0 (36.5–75.5)	7	41.2 (17.8–64.6)	0.346
Case report	15	60.0 (40.8–79.2)	12	70.6 (48.9–92.2)	0.482
Qualitative study	3	12.0 (− 0.7–24.7)	2	11.8 (− 3.6–27.1)	0.634
Other	1	4.0 (− 3.7–11.7)	0	0	0.999
None	2	8.0 (− 2.6–18.6)	1	5.9 (− 5.3–17.1)	0.727

*BPI* Brief Pain Inventory, *ESAS* Edmonton Symptom Assessment Scale, *MDASI* MD Anderson Symptom Inventory, *NRS* numeric rating scale, *PC* palliative care, *PROM* patient-reported outcome measure, *RCT* randomized controlled trial

Only a few hospitals offered PC research programs, more of which were reported in cancer hospitals (*n* = 17, 17.2%) than in tertiary general hospitals (*n* = 25, 9.3%; *p* < 0.001). Often, PC research teams included fewer than 5 members, and in most cases extra funding for these research projects was not available; only about 20% of hospitals received funds for conducting PC research. Few abstracts reporting PC research were submitted to scientific conferences or for peer review in 2018: 56% of tertiary general hospitals and 58.8% of cancer hospitals submitted 1–5 abstracts for peer review.

Among the hospitals offering PC, the use of patient-reported outcomes (PROs) was reported by 39.8% of tertiary general hospitals and 45.1% of cancer hospitals. The most commonly used PRO tools were unidimensional measures, such as the numeric rating scale (> 33%), and multidimensional pain scales (> 25%) such as the Brief Pain Inventory. Use of multisymptom assessment tools, such as the MD Anderson Symptom Inventory (~ 16%) and Edmonton Symptom Assessment Scale (~ 11.5%), was limited.

## Discussion

To our knowledge, this is the first national survey of current PC practice for adult patients with cancer in China. Most hospitals reported that PC service was available; however, only approximately 1 in 3 cancer hospitals and 1 in 6 tertiary general hospitals reported having a PC department or specialist. As shown in this survey, inpatient and outpatient palliative care services in China were mostly provided by oncologists instead of PC specialists. Cancer hospitals were significantly more likely than tertiary general hospitals to have inpatient beds for PC, outpatient PC clinics, and PC educational and research programs. Our findings highlight opportunities for further development of PC clinical, educational, and research programs in China and underscore the role of cancer hospitals as models of PC.

Importantly, randomized controlled trials have shown that specialized PC ranging from basic symptom management to goals-of-care discussions [11, 12], when added to usual oncologic care, can improve quality of life, symptom control, patient and caregiver satisfaction, quality of end-of-life care, and possibly survival [13]. From a practical standpoint, it is important to recognize that oncologists and nursing staff often have very limited clinic time, making it difficult to address supportive care issues comprehensively [14, 15]. They may also lack the interest, knowledge, screening tools, and interdisciplinary teams needed to provide state-of-the-art supportive care. Finally, patients may be reluctant to bring up their supportive-care needs during a visit with their oncologists, due to lack of knowledge about palliative care, a cultural taboo around death, or factors related to patient-clinician conflict [7]. Possible reasons for a family’s inability to continue to care for the patient include severe symptom distress, caregiver burden or illness, and lack of broader family support.

It is encouraging that cancer hospitals in China were more likely than tertiary general hospitals to have PC specialists and infrastructure. However, much more work is needed. First, PC needs to be recognized as a specialty in China to standardize training and recruit health professionals; at present, no PC license or certification programs are available. (As referenced in this paper, a “PC specialist” is anyone participating in an institutional PC program in China.) Second, the availability of PC should be considered as part of accreditation for cancer hospitals, similar to the American College of Surgeons Commission on Cancer program [16]. Third, hospitals need to invest more resources into PC. This is particularly important, given the aging population in China and the greater proportion of patients living with cancer as a chronic illness [1, 17]. Fourth, incorporating specialized PC into oncology treatment guidelines may help to standardize practice [18]. Fifth, national policies and campaigns may significantly stimulate the interest in and awareness of supportive care [2, 19]. China has implemented a number of such policies over the past few decades. It has also established several demonstration projects as regional centers of clinical excellence, education, and research [5].

Each branch of PC serves an important role [2]. We found that inpatient consultation teams were most common (approximately 50%), followed by inpatient beds for PC (approximately 40%), and outpatient clinics (> 30% of hospitals). In addition, more than one-third of hospitals with a PC service also offered inpatient consultations on weekends. These services were relatively urgent or severe, and referrals were often to be addressed timely, suggesting opportunities to further develop these programs [20].

Cancer pain and symptom management has been a featured effort of PC practice in China in the past 2 decades. Initially, PC training in China was primarily pain management—a trend that remains even today. National policy for opioid access varies: for cancer patients, every prescription of strong opioids is limited to 14 days; for non-cancer patients, opioid treatment cannot extend beyond 8 weeks. Although not yet standard care in China, the use of established PRO tools for pain assessment is growing, as reflected by our finding that 40%-45% of hospitals with PC available reported using these validated PRO tools in their PC practice. The Brief Pain Inventory was culturally adapted and psychometrically validated for use in China in 1995 [21] and has since been used in professional training programs around the country [5]. Both the Edmonton Symptom Assessment Scale and the MD Anderson Symptom Inventory also have been psychometrically and linguistically validated in Chinese [22, 23]. Multisymptom management consensus or guidelines have been established by professional societies [24, 25].

No national fund has been established for PC research, such as studies on pain management, psychosocial support, social work, and nutrition programs. Professional societies and local governments often provide financial support for establishing PC practice but not for research. We found that few Chinese hospitals had large clinical PC programs with adequate infrastructure and staffing to support trainees and research investigations, although cancer hospitals were more likely than tertiary general hospitals to have PC education and research programs. This highlights the role of cancer hospitals as models of care for disseminating PC knowledge and advances in China [26]. Formalizing PC as a medical specialty, establishing dedicated funding for PC training and research, and developing government policies to stimulate PC development are urgently needed [27]. Collaboration with other countries that have better-developed PC programs may also accelerate the development of PC training programs and research in China. Although we did not specifically assess palliative care training for medical oncology fellows in China, to the best of our knowledge, limited formal education is offered at this time, and no mandatory rotation to a PC unit is required due to limited PC services. Given the importance of PC, this represents a key area for future development.

Our study had limitations. First, the response rate of this study was 48% by cancer hospitals in China, however, this is the whole sample survey design; on another hand, for matched tertiary general hospitals, the response rate was 60.5%, which reached our expected number (at least 257 hospitals would need to be surveyed, actual *n* = 268) to achieve 80% power to test the primary outcome. The hospitals without PC services may have been less likely to participate, although we did not have data to compare PC availability between responders and non-responders. Second, because PC is not yet an accredited specialty in China, most of the respondents were medical oncologists describing their primary PC practice, rather than PC specialists. Third, we only surveyed cancer hospitals and tertiary general hospitals, which represent some of the top hospitals in China with well-developed academic programs and sufficient governmental resources. Thus, our findings probably represent the best possible conditions for PC practice in China. The Chinese government intends that delivery of PC be consistent nationwide. Our findings, although based on a different methodology, are generally consistent with those of the World Atlas of Palliative Care indicating that China is at the preliminary stage of PC integration [28]. Further research is needed to assess variations in PC access among different regions in China and to examine community-based PC practices, PC for pediatric patients with cancer or hematological diseases, and end-of-life care.

In summary, our results indicate that PC is emerging in China, with some particularly encouraging developments at cancer hospitals. This real-world evidence provides an important benchmark for PC clinical, educational, and research programs. Given the significant benefits associated with specialized PC, [29–31] our findings call for greater investment in PC infrastructure in China at the departmental, institutional, regional, and national levels.

### Supplementary Information

**Additional file 1.** Additional items for China professional survey on palliative care.

## Data Availability

The data underlying this article could be shared upon reasonable request to the corresponding author.
