# Supplementary material for: National survey on the availability of oncology palliative care services at tertiary general and cancer hospitals in China
Source: BMC Palliat Care. 2023 Sep 28;22:144. doi: 10.1186/s12904-023-01259-5 (PMC10536755; doi:10.1186/s12904-023-01259-5)
Supplement: Supplementary file 1 — Additional file 1. Additional items for China professional survey on palliative care. [file 12904_2023_1259_MOESM1_ESM.doc]

**Supplementary Table 1**

**Additional Items for China Professional Survey on Palliative Care**

2.8 Do you and your team routinely use Patient Reported Outcomes (PROs) to assess symptoms, function and efficacy of treatments?

口Yes (please continue answering) 口No (please jump to question 2.9)

2.8.1 Which of the following PRO scales are used in your clinical practice? (Can be multiple selection)

口Single-dimensional pain assessment, such as digital pain grading (NRS), etc

口Multidmensional pain assessment, such as Brief Pain Inventory (BPI),etc

口Multisymptom Assessment Scale, such as MD Anderson Symptom Inventory (MADASI)

口Palliative Care Scale, such as Edmonton Symptom Assessment Scale (ESAS)

口Other tools, please add underline:

2.10 The number of palliative care services in your hospital:

| Staff | 0 | 1-5 | 6-10 | 11-25 | 26-50 | ＞50 |
| --- | --- | --- | --- | --- | --- | --- |
| Ward nurses (inpatient) |  |  |  |  |  |  |
| Outpatient nurses (outpatient) |  |  |  |  |  |  |

| Staff or faculties | 0 | 1 | 2-5 | 6-10 | ＞10 |
| --- | --- | --- | --- | --- | --- |
| Chaplain |  |  |  |  |  |
| Nutritionist |  |  |  |  |  |
| Middle medical staff (senior nurse or assistant physician) |  |  |  |  |  |
| Palliative medicine specialist |  |  |  |  |  |
| Rehabilitation physician |  |  |  |  |  |
| Traditional Chinese Medicine doctor |  |  |  |  |  |
| Clinical pharmacist |  |  |  |  |  |
| Psychiatrist |  |  |  |  |  |
| Psychologist |  |  |  |  |  |
| Pain specialist/anesthesiologist |  |  |  |  |  |
| Social worker |  |  |  |  |  |
| Other |  |  |  |  |  |

2.13 Does palliative care program in your hospital requires physicians to have a national certified palliative care professional certificate?

口Yes 口No

2.14 Does palliative care program in your hospital requires nurses to have a national certified palliative care professional certificate?

□ Yes □ No

8.5 Please explain the approximate number of abstracts your team submitted at academic conferences in 2018:

Academic scientific conferences in oncology (e.g. CSCO, Oncology Branch of Chinese Medical Association, etc.) □ 0 □ 1-2 □ 3-5 □ 1-6-10 □> 10

Palliative care conferences (e. g. CRPC, etc.) □ 0 □ 1-2 □ 3-5 □ 6-10 □> 10

Other _______□ 0 □ 1-2 □ 3-5 □ 6-10 □> 10

8.6 Please explain the approximate number of papers published by your team in 2018:

Oncology journals □ 0 □ 1-2 □ 3-5 □ 6-10 □> 10

Palliative Care journals □ 0 □ 1-2 □ 3-5 □ 6-10 □> 10

General Medicine journals □ 0 □ 1-2 □ 3-5 □ 6-10 □> 10
